# Sensing CA 15-3 in point-of-care by electropolymerizing *O*-phenylenediamine (oPDA) on Au-screen printed electrodes

**DOI:** 10.1371/journal.pone.0196656

**Published:** 2018-05-01

**Authors:** Rui S. Gomes, Felismina T. C. Moreira, Ruben Fernandes, M. Goreti F. Sales

**Affiliations:** 1 BioMark/ISEP, School of Engineering, Polytechnic Institute of Porto, Porto, Portugal; 2 CEB, Centre of Biological Engineering, Minho University, Braga, Portugal; 3 Faculty of Engineering, Porto University, Porto, Portugal; 4 Pharmacology Centre and Biopathology Chemistry, Medicine Faculty, Porto University, Porto, Portugal; 5 Superior Health School, Polytechnic Institute of Porto, Porto, Portugal; Ludwig-Maximilians-Universitat Munchen, GERMANY

## Abstract

This work presents an alternative device for cancer screening in liquid biopsies. It combines a biomimetic film (i) with electrochemical detection (ii). The biomimetic film (i) was obtained by electro-polymerizing amine-substituted benzene rings around a CA 15–3 target. This protein target was previously adsorbed on a gold (Au) support and incubated in charged monomers (4-Styrenesulfonate sodium and 3-Hydroxytyraminium chloride). The protein was further eliminated by enzymatic activity, leaving behind vacant sites for subsequent rebinding. Electrochemical detection (ii) was achieved on an Au working electrode, designed on commercial screen-printed electrodes. Raman spectroscopy, atomic force microscopy and ellipsometric readings were used to follow the chemical modification of the Au surface. The ability of the material to rebind CA15-3 was monitored by electrochemical techniques. The device displayed linear responses to CA15-3 ranging from 0.25 to 10.00 U/mL, with detection limits of 0.05 U/mL. Accurate results were obtained by applying the sensor to the analysis of CA15-3 in PBS buffer and in serum samples. This biosensing device displayed successful features for the detection of CA 15–3 and constitutes a promising tool for breast cancer screening procedures in point-of-care applications. Moreover, its scale-up seems feasible as it contains a plastic antibody assembled *in situ*, in less than 1 minute, and the analysis of serum takes less than 30 minutes.

## Introduction

Breast cancer is the most common form of cancer in women and is one of the main causes of death worldwide [1]. Advances in medical treatments have emerged to improve current therapies, but early detection remains a key element for the successful outcome of the disease [2, 3]. Early detection may be feasible by tracking a cancer biomarker that may circulate in biological fluids, allowing less invasive procedures. Cancer biomarker is a substance abnormally expressed in tumour tissue compared with normal tissues [4, 5]. It may include DNA or RNA modifications, protein expression and presence in human fluids, etc.[6]. In this context, CA 15–3 has been found in the serum of more than 70% of patients with breast cancer [7] and, in lower concentration, in patients with other cancer disease that are not breast related. CA 15–3 is also present in almost 70% of metastatic breast cancer events [8]. Therefore, CA15-3 is a promising biomarker for early detection of breast cancer [1, 7].

Some conventional immunoassay methods, such as the enzyme-linked immunosorbent assay [9], radioimmunoassay [10], fluorescence immunoassay [11], electrophoretic immunoassay [12] and high-performance liquid chromatography (HPLC) [13] have been described in the literature for CA15-3 protein detection. However, these methods are complex, high time-consuming, expensive, labor-intensive and unsuitable for POC applications. Therefore, it is of interest to investigate further for accurate, rapid and simple alternative methodologies for the determination of CA 15–3.

Moreover, the increasing demand for ultrasensitive determination of tumor markers is pushing for novel detection technologies. In this context, electrochemical devices are becoming a promising alternative for screening cancer biomarkers due to their low price, simple operation and POC feasibility. Several electrochemical immunoassays have also been reported in literature [12, 14–16], offering attractive features as low cost and portability. Most of these use biological elements as enzymes and antibody/antigen interaction for CA 15–3 detection [12, 14, 17]. Such biological elements offer little stability, require highly complex production schemes and involve high cost. Artificial counterparts of these “natural” antibodies have emerged to overcome these disadvantages, offering robustness, resistance to temperature and pressure changes, and low cost [18–20].

Several strategies have been reported to integrate molecular imprinting (MI) technology in biosensors ([21] [22, 23]. Conventional bulk imprinting has been reported [24] [20, 25–27], along with new improved formats [28–32]. Among these, electropolymerization is an attractive alternative [31, 33–36]. In this, the films are synthetized *in situ*, within few seconds/minutes and involving very little amount of reagents [36]. Electroactive monomers used for this purpose are aromatic rings with different chemical functionalities. Among these, amine functions prevail, mostly due to their easy interaction with proteins, through hydrogen bonding. In this regard, MI sensors including polyaminophenol (oAP, [33], poly(*o*-phenylenediamine) (PoPDA) [18], and polyaniline [18] have been reported in the literature. In the case of PoPDA based compounds, polymers with controlled thicknesses, ranging within 10–100 nm [37], due to a self-limiting growth, were *in situ* electropolymerised and were easily regenerated after use [38, 39]. In addition to this, the degree of polymeric reticulation and porosity are important features in terms of rebinding recognition, affecting the selective/specific recognition of an imprinted protein. Considering PDA, such features could be an outcome of the positions of the amine functions on the benzene ring.

Thus, it would be interesting to compare different monomers and identify which position would benefit more the formation of a suitable protein imprinted polymer for a given protein. This hypothesis was tested herein by comparing systematically similar monomers with chemical functions in different positions. Aniline (a single amine function), oPDA (two amines in 1,2 positions) and pPDA (two amines in 1,4 positions) were selected for this purpose. The intermediate position, *m*-phenylenediamine (two amines in 1,3 positions) was disregarded, because preliminary studies indicated that it did not produce a stable material. From a regular mechanism perspective, it is clear that the amine is a strong activator of the aromatic ring towards electrophilic substitution, thereby indicating that the dominant substitution positions are *orto* and *para*.

In In addition to this, it would be important to understand the effect of charged species at the rebinding site. When the polymer is tailored with a single monomer, the rebinding recognition is mostly established by the format of the rebinding site, besides the electrostatic interactions established throughout the external surface of the polymer (including other sites than the rebinding site). If a charged species is added into the rebinding site (and only at this site), the recognition may be established by format and differentiated electrostatic interactions. This concept, previously tested before and named SPAM [40], is tested herein by preparing imprinted materials with charged species.

Thus, this work describes the electropolymerization of films made with amine-substituted benzene rings as monomers (Aniline, oPDA and pPDA) and specific charged monomers (4-Styrenesulfonic Acid Sodium Salt, negatively charged; and dopamine, or 3-Hydroxytyraminium Chloride, positively charged) for generating oriented protein imprinting materials for CA15-3 detection. The protein CA 15–3 was adsorbed onto a gold surface, surrounded by the growing polymeric matrix, followed by protein removal with a proteolytic enzyme (cleaving peptide bonds and subsequently destroying the structure of imprinted proteins or peptides). A control material, named non-imprinted (NI) polymer was synthesized by the same way but without the template. The resulting biosensor was evaluated by cyclic voltammetry (CV), electrochemical impedance spectroscopy (EIS) and square wave voltammetry (SWV) techniques and further applied to the analysis of biological sample. This biosensor showed to be a promising tool for screening CA15-3 in point-of-car (POC), offering simplicity of fabrication, low response time, low cost, and good sensitivity/selectivity.

## Materials and methods

### Apparatus

The electrochemical measurements were conducted with a potentiostat/galvanostat from Metrohm Autolab and a PGSTAT302N, equipped with a FRA2 module and controlled by Nova 10.1 software. Raman measurements were performed using a Thermo Scientific DXR Raman microscope system with a 100 mW 532 nm excitation laser. Ellipsometry measurements were performed using a J.A.Woollam Co, Inc M/2000V model ellipsometer with a 370–1000 nm spectral range and 50 W QTH lamp equipped with an EC/400 electronics control module and controlled with CompleteEASE v4.92 software. Atomic force microscopy (AFM) measurements were performed using Veeco Metrology Multimode/Nanoscope IVA. Au-SPEs were purchased from DROPSENS (DRP-C220AT), having working and counter electrodes made of gold and reference electrode and electrical contacts made of silver. The diameter of the working electrode was 4 mm. The Au-SPEs were interfaced with the potentiostat by means of a compatible switch box, from BioTID/Porto-Portugal.

### Reagents

All chemicals were of analytical grade and de-ionized water (conductivity <0.1 μS/cm) was employed. The following reagents were obtained from Sigma-Aldrich: Breast Tumour Antigen from human fluids (CA15-3), myoglobin (Myo), o-phenylenediamine (oPDA), p-phenylenediamine (pPDA), 3-hydroxytyraminium chloride (Dopamine, DPM) and trypsin. Potassium hexacyanoferrate III (K_3_[Fe(CN)_6_]), L-ascorbic acid (AA) and potassium hexacyanoferrate II (K_4_[Fe(CN)_6_] trihydrate were obtained from Riedel-De Haën. 4-Styrenesulfonic Acid Sodium Salt (SA) was obtained from Alfa Aesar. Potassium di-hydrogenphosphaste (KH_2_PO_4_) and Sodium chloride (NaCl) were obtained from Panreac. Potassium chloride (KCl) was obtained from Merck. Glucose (Glu) was obtained from Fisher BioReagents. Creatine kinase isoenzyme (CK-MB) was obtained from ERM-European Reference Materials. Creatinine (Crea) and bovine serum albumin (BSA) were obtained from Amresco and Aniline from Analar Normapur.

### Solutions

Stock solutions of 100 U/ml CA15-3 were prepared in PBS buffer (1.0×10^−2^ mol/L, pH 7.36). Less concentrated standards were obtained by accurate dilution of the previous solution in the same buffer. Electrochemical assays were performed with 5.0×10^−3^ mol/L K_3_[Fe(CN)_6_] and K_4_[Fe(CN)_6_] in PBS 1.0×10^−2^ mol/L, pH 7.36. PBS was composed of NaCl (136.90 mM), KCl (2.68 mM), Na_2_HPO_4_ (12.54 mM) and KH_2_PO_4_ (1.76 mM), with pH set to 7.36. To simulate artificial human serum, a solution od with NaCl (120.00 mM) and NaHCO_3_ (20.00 mM) with pH set to 7.4 was used instead of buffer.

### Design of the biomimetic sensor on the Au-SPE

**Fig 1:**
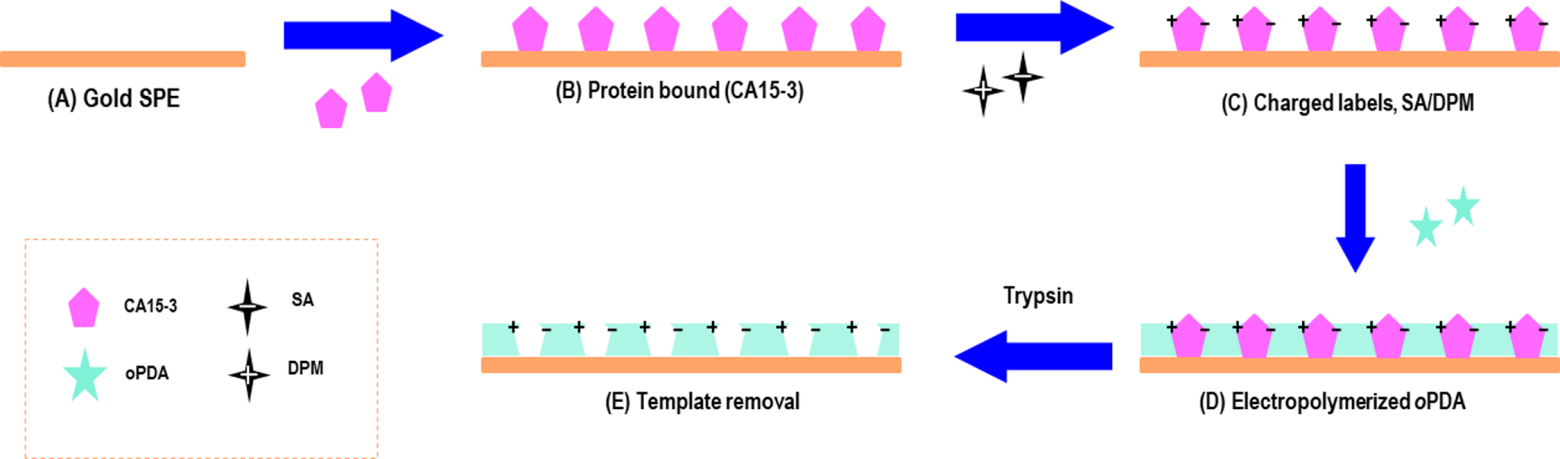
Schematic representation of the synthesis of the imprinted material. (A) Au-SPE; (B) CA15- 3 adsorption on Au-SPE surface; (C) Charged monomers labelling; (D) Electropolymerization of PDA; and (E) binding site formation by template removal with Trypsin.

The gold surface of the working electrode (Au-SPE) was cleaned with ethanol. CA15-3 (100 U/mL) was prepared in PBS, solution pH 7.36, and incubated in the Au-SPE for 90 min at room temperature (Fig 1B). The CA15-3/Au-SPE was washed with PBS and then was incubated overnight with SA (0.1 mM) and DPM (0.1 mM) solution, also prepared in PBS and subsequently washed with PBS (Fig 1C).

The imprinted material was obtained by electropolymerization of a monomer 5 mM solution in PBS on CA15-3/Au-SPE surface [40] (Fig 1D). The polymerization was carried out by CV measurements with potential range between -0.45 and 0.80V, scan rate of 100 mV/s, for 10 cycles. CA 15–3 was removed from the polymeric matrix by incubation is trypsin solution for 90 min, at 36°C. The film was finally electrochemically cleaned with PBS buffer in order to remove protein fragments and adsorbed trypsin, and finally rinsed with DI water. This was performed by CV scanning, between -0.30 and 0.30 V, for 10 cycles (20 stop crossing) (Fig 1E). A negative control was also produced excluding the protein adsorption step on Au- SPE from the procedure and is named non-imprinted materials (NI)

### Morphological analysis of the guest-polymer

Relevant chemical/physical data of the synthetic materials was obtained by surface analysis using FTIR and Raman spectroscopy, ellipsometry and AFM. AFM surface characterisation studies were conducted only on planar gold surfaces (100 nm gold chips in a 25 nm Silica Dioxide wafer with 2 nm Ti), because the surface of the SPEs was too rough. AFM studies were applied to gold, MIP/-/Au, MIP/CA15-3/Au, and NIP/Au surfaces, without any prior treatment.

FTIR studies were done by direct analysis of the solids on an ATR diamond crystal support; the samples selected for this study were Au-SPE without any prior treatment, MIP/-/Au, MIP/CA15-3/Au, and NIP/Au surfaces. Raman studies were obtained by direct observation of the sample on the confocal microscope (of the Raman equipment); the samples considered for this study were MIP/-/Au after incubation in CA15-3 0.1 U/ml and 5.0 U/ml, and NIP/Au-SPE surfaces.

### Electrochemical procedures

CV and SWV measurements were conducted in 5.0 mmol/L of [Fe(CN)_6_]^3-^ and 5.0 mmol/L of [Fe(CN)_6_]^4-^, prepared in PBS buffer (1.0×10^−2^ mol/L, pH 7.36). For CV assays, the potential was scanned from -0.7 to +0.7 V, at 50 mV/s. For SWV studies, potentials were changed from -0.3 to +0.5 V, at a frequency of 50Hz with a step height of 150 mV. All assays were conducted in triplicate.

EIS assays were performed with the same redox couple [Fe(CN)_6_]^3-/4-^ with open potential circuit (OCP), using a sinusoidal potential perturbation with amplitude of 0.01 V and a number of frequencies equal to 50, logarithmically distributed over a frequency range of 0.1–100 kHz. The impedance data were fitted with commercial software Nova. The Randles equivalent circuit is used to fit all the electrical parameters of the EIS results, reflecting the electrochemical proprieties of the electrode-solution interfaces with the occurrence of Faradaic current diffusion transport [41, 42]. The Nyquist plot of a Randles cell consists of a semicircle. At high frequencies the impedance of Cdl is very low; at very low frequencies the impedance of Cdl becomes high, and thus, the measured impedance tends to charge transfer resistance and solution resistance (Rct + Rs). The diameter of the semicircle is identical to the charge-transfer resistance [43]. The diffusion was assigned to the Warburg element (W).

The calibration curve was performed by SWV and EIS measurements. Readings were made for MIP with and without charged labels and for NIP materials, with each assay performed at least 3 times. Calibration curve was achieved by 15 minutes incubation of the sensor with consecutive concentrations of CA15-3, ranging 0.005 to 40.0 U/mL CA 15–3 solutions, prepared in PBS buffer.

CA15-3 assay in serum samples sample was performed by SWV measurements. CA15-3 was prepared in synthetic serum solution diluted 5 times, in a concentration range between 0.005 to 40.0 U/mL CA15-3.

Selectivity studies were performed in synthetic serum spiked with biological compounds present in physiological fluids. Spiked diluted serum samples were diluted 5 times in serum, with CA15-3 at constant 10 U/mL concentration. This solution was incubated in MIP for 15 min intervals with multiple SWV readings at the end of each interval.

## Results and discussion

### Design of the biosensor

**Fig 2:**
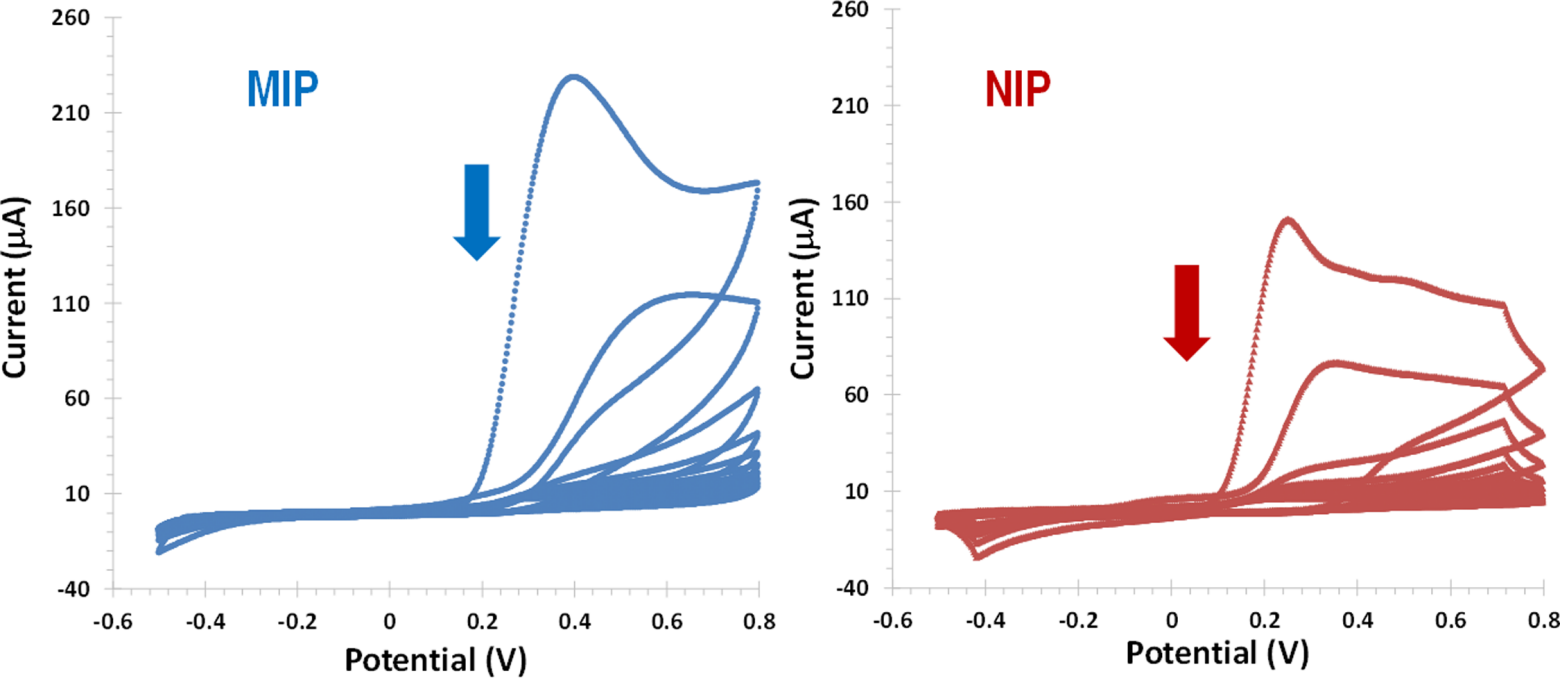
Electropolymerization of oPDA to produce MIP/CA15-3/Au-SEP (left) and NIP/–/Au-SPE (right). CV with 10 consecutives cycles in a potential range between −0.5 and +0.8 V and scan-rate 100 mV/s, in PBS buffer, pH 7.2.

Molecular imprinting was prepared by electropolymerizing different monomers (oPDA, pPDA and Aniline), using CA15-3 as a template molecule, by CV (Fig 2). The process consisted in 3 different stages (Fig 1) starting with CA15-3 adsorption on gold SPE surface, followed by the imprinting step by the monomer thin film formation on Au-SPE/CA15-3 surface, and terminated by the CA15-3 removal. Each of these stages was controlled by several electrochemical readings.

The CA15-3 imprinting started with its adsorption on the Au-SPE surface. This was made by casting a drop of the protein solution on the gold area. When applicable, charged monomers (positively charge 3-hydroxytyraminium, DPM, and negatively charged 4-Styrenesulfonic Acid Sodium Salt, SA) were incubated overnight.

Following, the electropolymerization of o-PDA on Au-SPE/CA15-3 surface was carried out by CV using 10 consecutives cycles in a potential range between -0.5 and 0.8 V and a scan rate 0.1 V/s, in PBS buffer of pH 7.2. During the electropolymerization step, the current decreased with the increasing of the number of cycles and the highest current was obtained in the first cycle. When 10 cycles were reached, the current density of the oxidation peak was smaller, meaning that the film was formed at electrode surface (Fig 2). Comparing to the NI film, the imprinted film displayed lower maximum current values, thereby confirming the presence of the adsorbed proteins on the surface. No reduction peak was observed during the electropolymerization confirming the growth of an insulator imprinted polymer on the Au-SPE electrode. The thickness of the film formed did not exceed 50 nm due to the self-limiting effect, caused by the low polymer film conductivity [44].

The protein was removed from its imprinted site with an unspecific protease (Trypsin), remaining the polymeric network of the artificial antibody. For this purpose the biosensor was incubated for 90 minutes with trypsin, at 36°C. This enzyme is highly active and stable with low cutting specificity and exhibits very wide cleavage specificity.

### Selection of the monomer to assemble the polymer matrix

**Fig 3:**
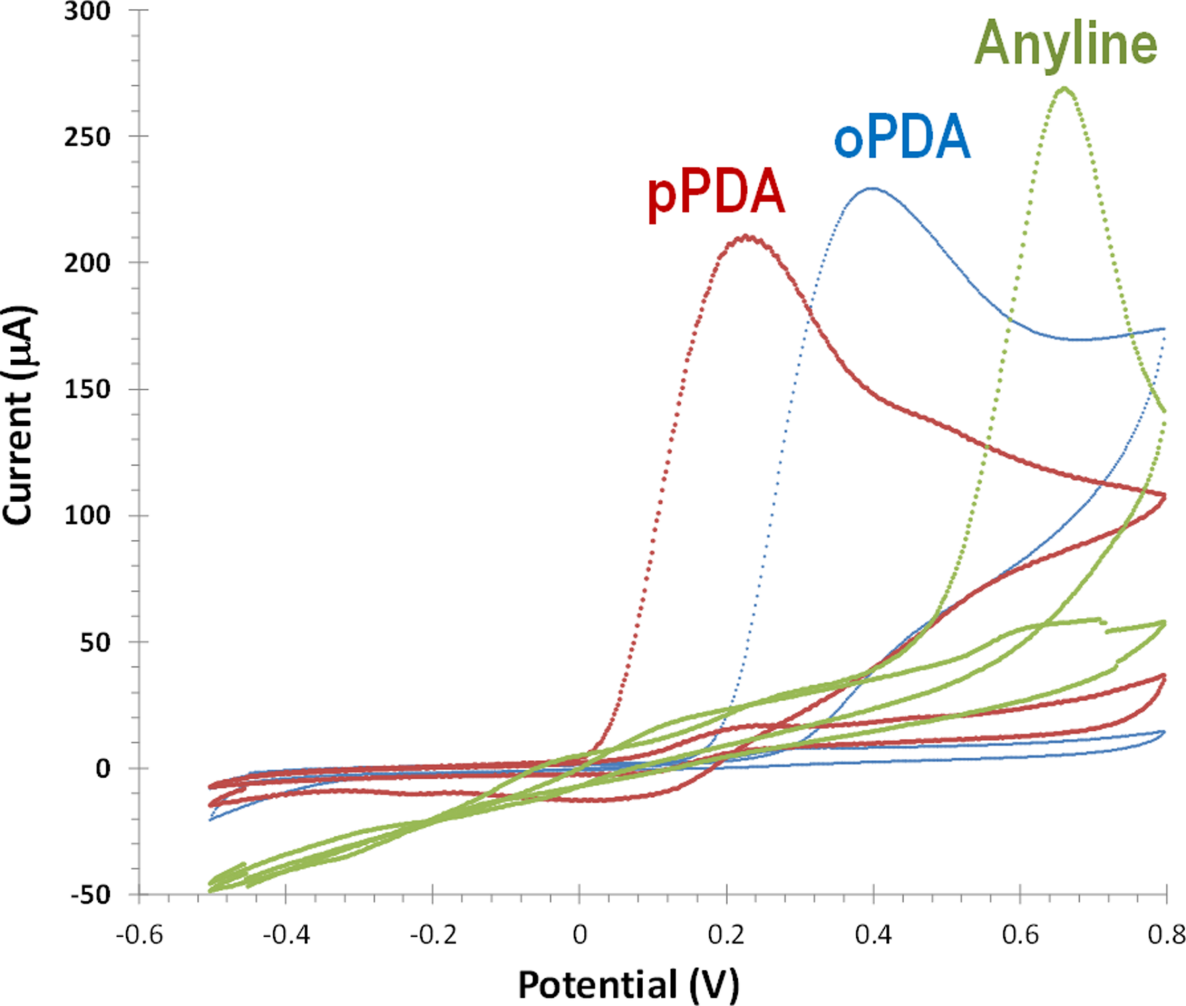
Electropolymerization of oPDA, pPDA and aniline on Au-SPE/CA15-3 surface. CV with 10 consecutives cycles in a potential range between −0.5 and +0.8 V and scan rate 100 mV/s, in PBS buffer, pH 7.2 (only first and last cycle are shown).

In this work, different monomers were tested to imprint CA15-3 within a polymeric matrix. These monomers were oPDA, pPDA and aniline. In general, electroactive functional monomers with amine functional groups linked to an aromatic ring are electropolymerizing to form non-conducting polymers under physiological conditions. PDA is a functional monomer for molecular imprinting synthesis, due to its ability to form compact/rigid polymer films, which offer hydrophilic, hydrophobic, basic, and other recognition sites [45]. In addition, it is possible to produce a thin and continuous film enabling to obtain a short response time of the imprinted biosensor. The electropolymerization was carried out in the same potential range (-0.2 to 0.8V) during 15 cycles, for all monomers. Non-conductive polymers were obtained with the three electroactive monomers. All monomers evidenced the formation of an oxidized radical species. As shown in Fig 3, the most easily oxidized monomer was oPDA (~0.2V), followed by pPDA (~0.4V) and by aniline (0.7V). Non-conductive polymers were obtained with the three electroactive monomers.

The resulting polymers were incubated in buffer and after in CA 15–3 standard solutions prepared in buffer to understand their typical electrical features (Figs 3 and S1). Overall, only the polymer obtained with oPDA displayed a stable electrical response after consecutive incubations in buffer and with the target protein. The higher stability of the poly(oPDA) was probably linked to the mechanism of radical polymerization involved, which shall be different from the other monomers, likely affecting the porosity and flexibility of the final polymer.

Overall, the monomer oPDA was selected for further studies, followed but the chemical and physical characterization of the resulting polymer.

### Follow-up of the physical/chemical changes occurring at the surface

#### Electrochemical follow-up

**Fig 4:**
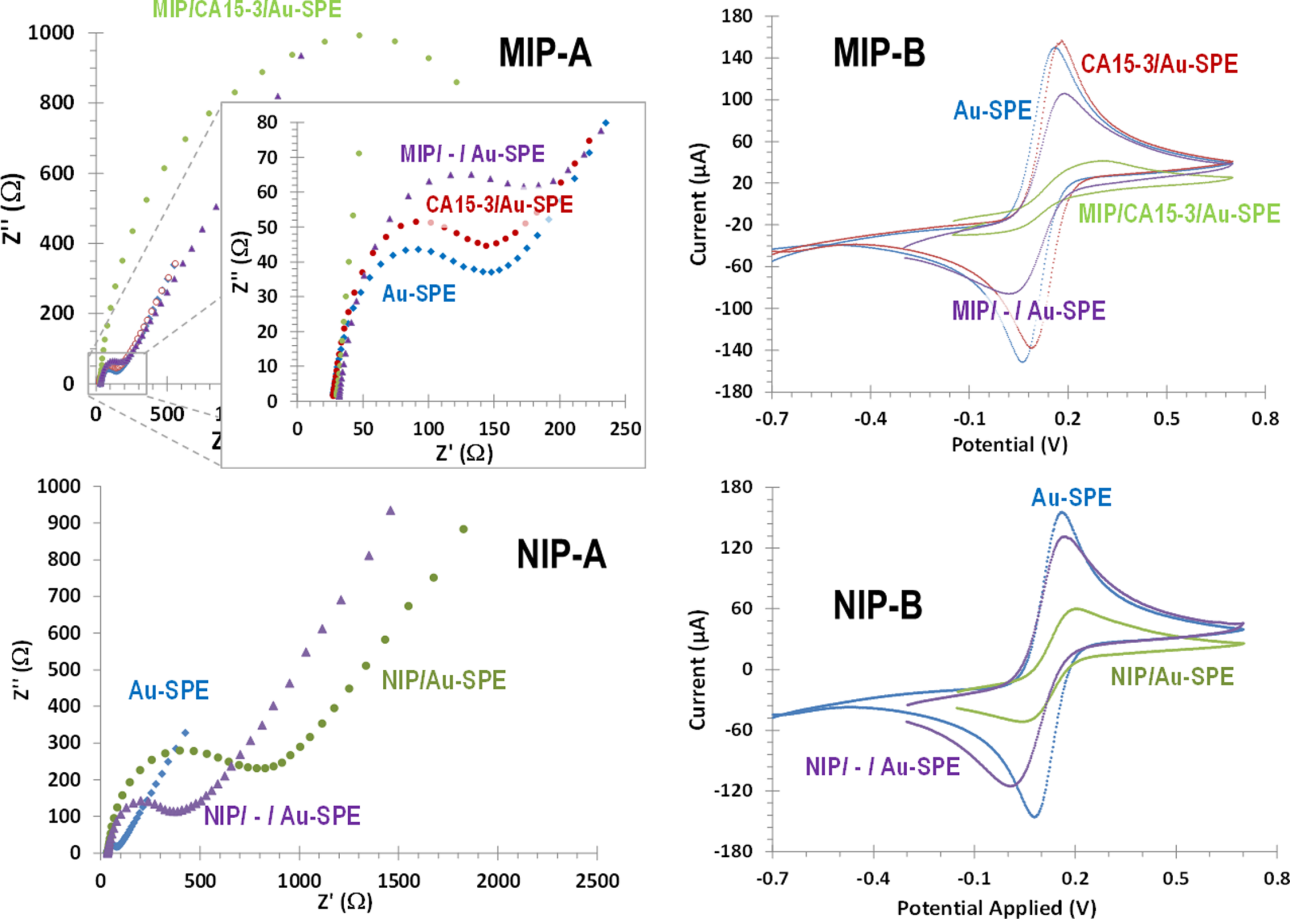
Electrochemical control of the subsequent modification steps of the Au-SPE to produce MIP (top) and NIP (bottom) films. Assessed in 5.0 mM [Fe(CN)6]^3−^ and 5.0 mM [Fe(CN)6]^4−^, in PBS buffer, pH 7.2, by EIS (A, Nyquist plots) and CV (B, cyclic voltammograms).

The electrochemical modifications made on the Au-SPE were followed-up by monitoring the changes in the electron transfer properties of a standard redox system. The redox couple [Fe(CN)_6_]^4-^/[Fe(CN)_6_]^3-^ was used for this purpose. The techniques used for to measure indirectly such alterations were EIS and CV assays. The obtained EIS spectra are presented as Nyquist plots (Fig 4A) showing a semicircle portion at high frequencies and a linear portion at low frequencies. The semicircle at higher frequencies corresponded to the electron-transfer-limited process, and the linear portion at lower frequencies represented the diffusion-limited process.

In EIS data, the presence of the CA15-3 protein on the surface of the Au- SPE after its adsorption was confirmed by an Rct increase, compared to the clean Au-SPE (Fig 4A). Next, the formation of the poly(oPDA) film introduced additional barriers to the electron transfer properties of the redox probe. This resulted in an extra increase in the electron transfer resistance, reflected by further substantial increase in Rct compared to the Au-SPE. This increase was much more evident in the MIP film, probably reflecting the presence of an insulating film plus the non-conductive CA 15–3. After protein removal, the resistance decreased substantially, suggesting that CA15-3 was successfully extracted from the polymer. Such decrease was much more evident in the MIP, because the protein was being extracted from it. In the NIP film, only a slight decrease in Rct was observed after trypsin incubation, probably related to the washout of small oligomer fragments form the surface.

CV assays are shown in Fig 4 and are consistent with the EIS results. The redox probe showed typical peak-to-peak potential separation values on the Au-SPEs. The subsequent adsorption of the protein promoted a slight peak decrease (more evident in the reduction peak) and a shift potential to higher values, thereby confirming the presence of an additional element on the Au-working electrode. After polymerization, the peak currents dropped to minimum levels, confirming the formation of an insulating layer on top of the Au-SPE surface. After template removal, the peak currents recovered, confirming the exit of CA 15–3 protein from the electrode surface. The NIP values showed a similar behaviour, except after polymerization, where the redox peaks of the probe remained evident.

#### RAMAN follow-up

**Fig 5:**
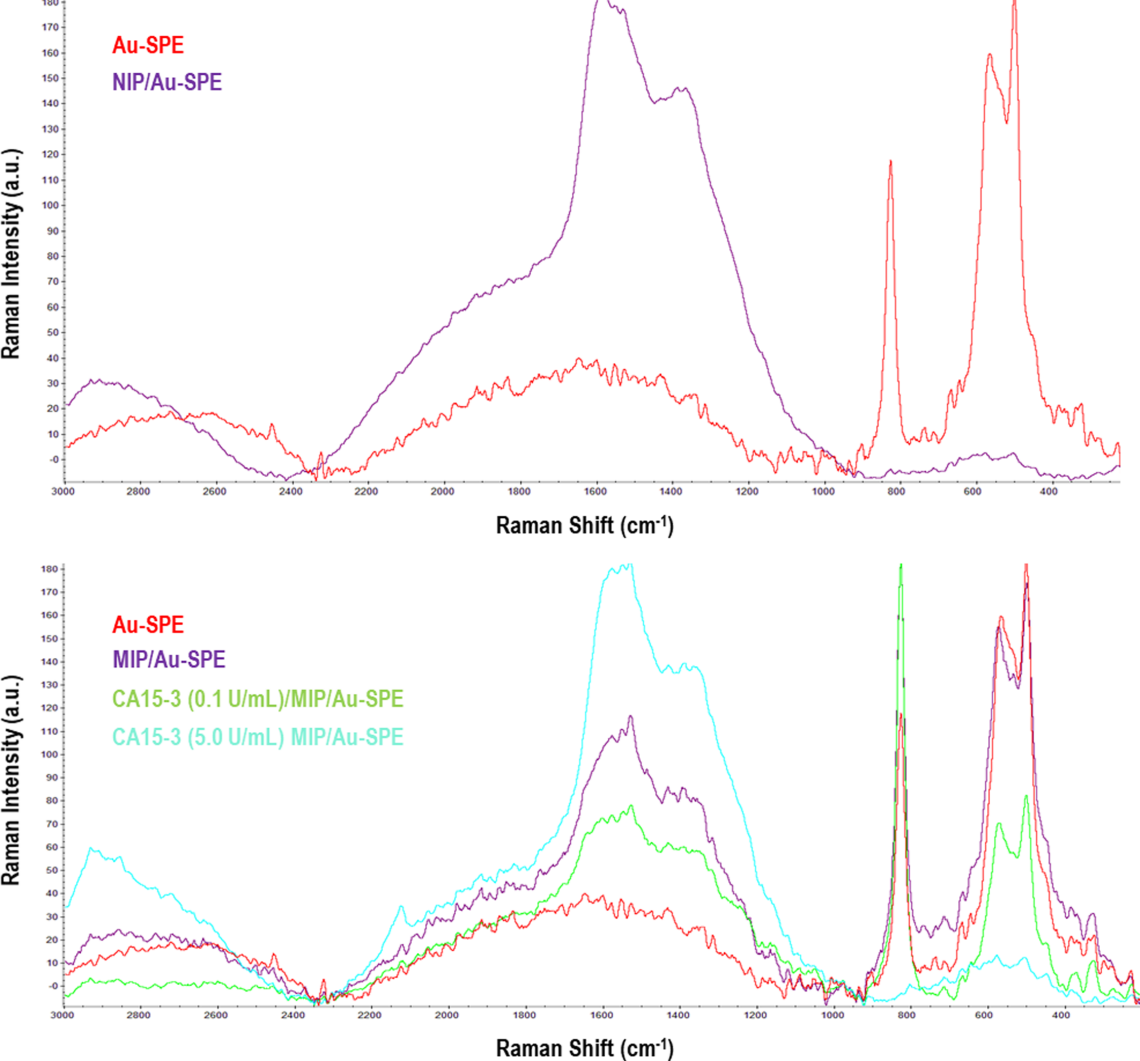
Raman spectra. Au-SPE, NIP/Au-SPE and MIP/−/Au-SPE with different concentration of CA 15–3.

Raman spectra were recorded for Au-SPE, MIP/-/Au-SPE, NIP/Au-SPE and MIP/-/Au-SPE surfaces with protein rebind (CA15-3, 0.1 and 5.0 U/ml) respectively. The typical images obtained are presented in Fig 5. Regarding NIP films, about the presence of the peaks at ~1400 cm^-1^ were linked to the C–N stretching vibration, having an intermediate single/double bond order and being coupled to quinoid-like rings [46]. In the region of around 1650cm^−1^, the Raman peak spectra was assigned to the carbon/carbon stretching vibrations of quinoid-like rings [47–49].

The MIP/-/Au-SPE showed the same bands as the NI material, with two additional peaks at about 800, 500 and 600 cm^-1^. These peaks were assigned to the Au-SPE electrode surfaces enabling the confirmation of the imprinted sites obtained after proteolithic treatment of the MIP/Au-SPE. The spectra of MIP/-/Au-SPE films incubated in protein showed an increase of the Raman peak at shift at about 1400 and 1600 cm^-1^ and a significant decrease at 800, 500 and 600 cm^-1^. This effect was concentration dependent, being more evident for higher concentrations. In one hand, the increase at the higher Raman shift range was linked to the increasing amount of the protein on the surface, while the decreasing values in the lower Raman shift revealed that the Au-SPE was covered by another material. The sensor incubated with higher concentration (5.0 U/ml) showed absence of the peaks (between 400 and 800 cm^-1^) suggesting that all cavities at the Au-SPE could be occupied by CA15-3 protein.

Overall, the RAMAN spectra confirmed the surface modifications and the presence of the imprinting sites of the sensor. Considering the great sensitivity of the Raman signal to different concentrations of CA 15–3 covering the gold, it is likely that the calibration of this biosensor could be follow-up by Raman spectroscopy, an approach not tested herein.

#### FTIR follow-up

**Fig 6:**
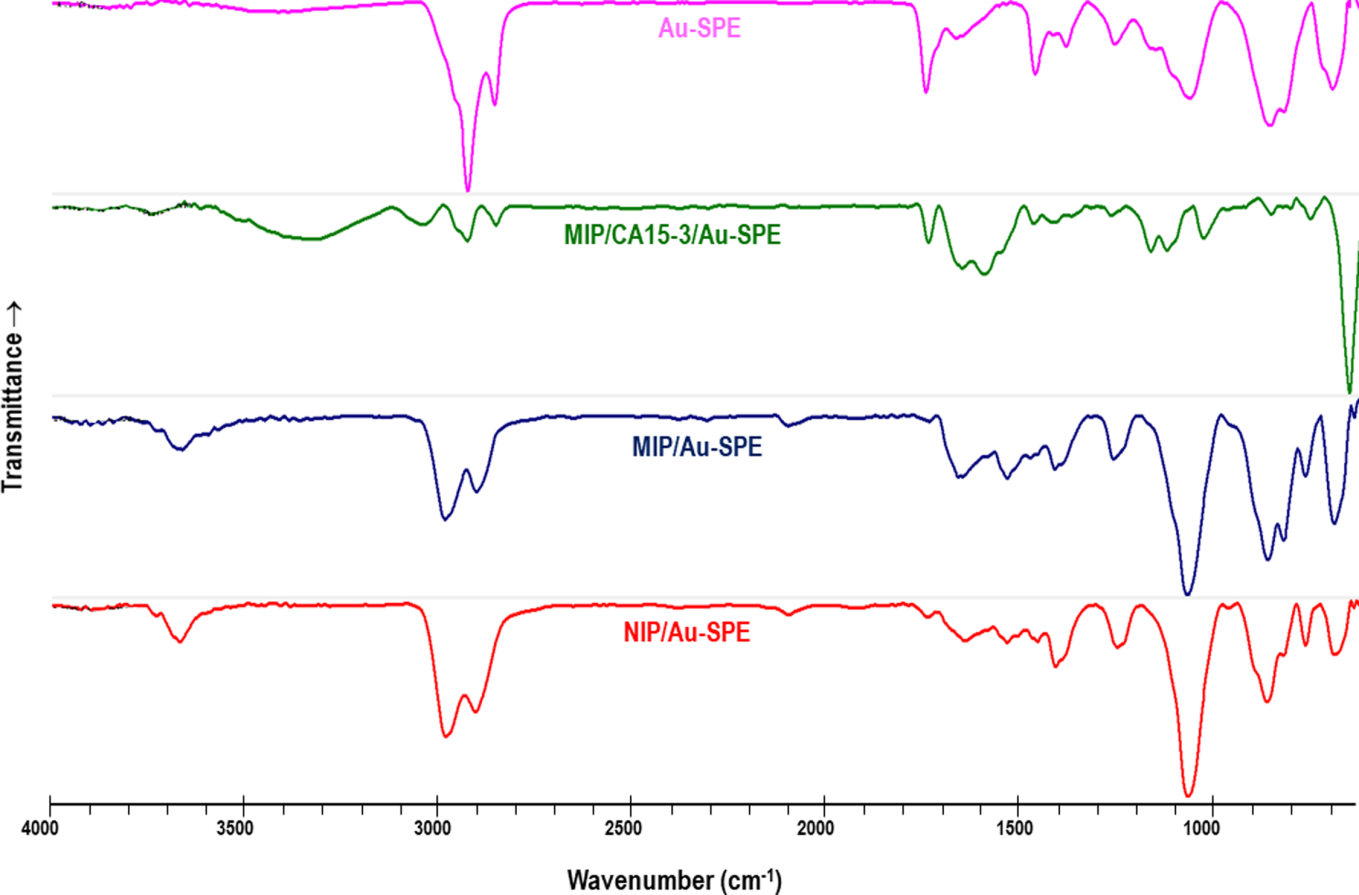
FTIR spectra. Au-SPE, MIP/Ca15-3/Au-SPE (before template removal), MIP/–/Au-SPE (after template removal), and NIP/–/Au-SPE.

FTIR analysis was used to confirm the surface modification on the Au-SPE surface. Data was collected within 4000–500 cm^-1^ wavenumber, at ambient temperature, for Au-SPE, MIP/CA15-3/Au-SPE, MIP/-/Au-SPE, NIP/Au-SPE sensors. The typical images obtained are shown in Fig 6.

Regarding the FTIR spectra of the Au-SPE, it confirms, in general, the presence of other organic compounds out coming from the gold ink deposited on the commercial SPE. The subsequent production of the MIP having the CA15-3 entrapped within the polymer, gives rise to an FTIR spectra that evidences clearly the presence of the protein at the surface. The chemical functions that are present in the protein, including N–H and O–H stretching from amine and carboxylic acids within the peptide moiety and from the glycosylated fraction of the protein, are probably responsible for the wide band centred at 3306 cm^-1^. Moreover, the absorption band ranging 1600–1700 cm^-1^ accounts the C = O stretching vibrations from the several amino acid residues within the protein.

As expected, the polymeric materials MIP (after template removal) and NIP showed several similar peaks, indicating their similar chemical composition. In general, the most significant peaks among the spectra revealed the functional groups present within the polymeric matrices. The small peak at about 3600 cm^-1^ was assigned to the C–N stretching vibrations of the polymeric network. The C–H aromatic/aliphatic stretching was evidenced near 2924 and 2850 cm^-1^, the C = C aromatic stretching at about 1600 and 1500 cm^-1^; the C–H deformation at about 1400 cm^-1^; the C–N stretching at about 1250 cm^-1^; and = C–H deformation at 1050 cm^-1^ (probably shifted from the normal position at ~1000 cm^-1^ due to the acceptor substitute groups underlying in the final polymeric network).

A final observation is related to the fact that the bands at 853 and 817 cm^-1^ in the MIP/CA15-3/Au-SPE disappeared after template removal. These bands were characteristic of the clean Au-SPE, revealing that the gold surface was exposed again after the protein removal. Overall, this confirmed the formation of the rebinding cavities on the MIP/-/Au-SPE sensor.

#### AFM follow-up

**Fig 7:**
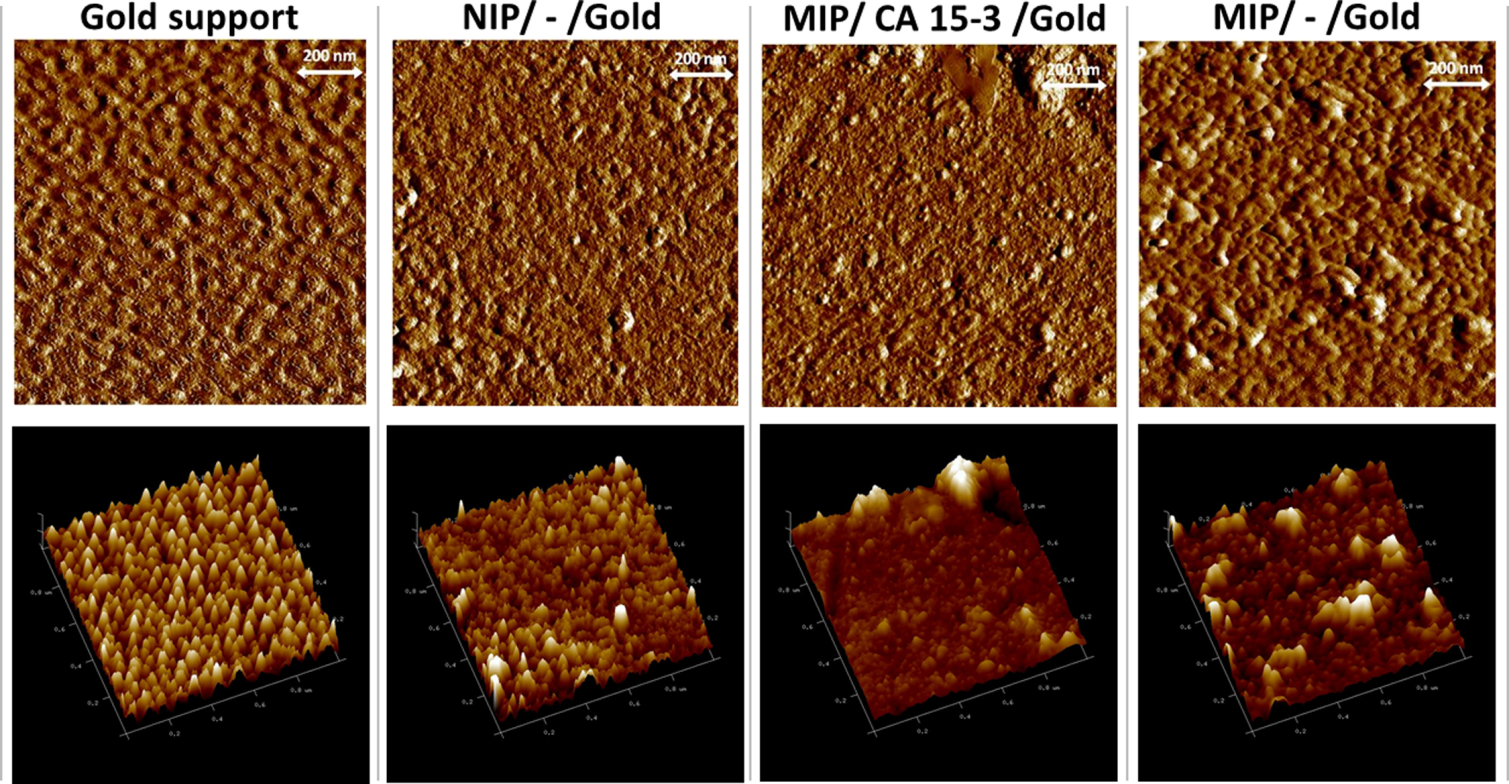
AFM images. Planar gold, NIP/–/gold, MIP/CA15-3/gold and MIP/–/gold.

The superficial analysis with AFM was performed on the same materials, but prepared on flat gold-surface surfaces. The surface of the Au-SPEs was too rough to allow withdraw some information about the subsequent modifications made on top of it. The topographic images of planar gold, NIP/gold, MIP/CA15-3/gold and MIP/-/gold sensors are shown in Fig 7 and suggested several structural differences between the materials.

The clean gold presented the smallest roughness levels under AFM observation. The addition of a polymeric layer on this gold support increased the roughness of the overall material. From a 2D view, it was clear that the typical profile of the clean gold was lost. The adsorption of the CA15-3 followed by electrical polymerization gave rise to an apparently more plane surface, but containing globular structures on top, of huge size compared to the polymer roughness. These globular structures were no longer evident after treatment with trypsin, thereby confirming the exit of the protein. In general, the roughness of the MIP/-/Au-SPE was about 15 nm which was a higher value than the NIP material, accounting the presence of the rebinding cavities.

Overall, the AFM data obtained along the different stages of the material modification was consistent with the previous data, of electrochemical or chemical nature.

### Selection of the charged monomer to improve rebinding

**Fig 8:**
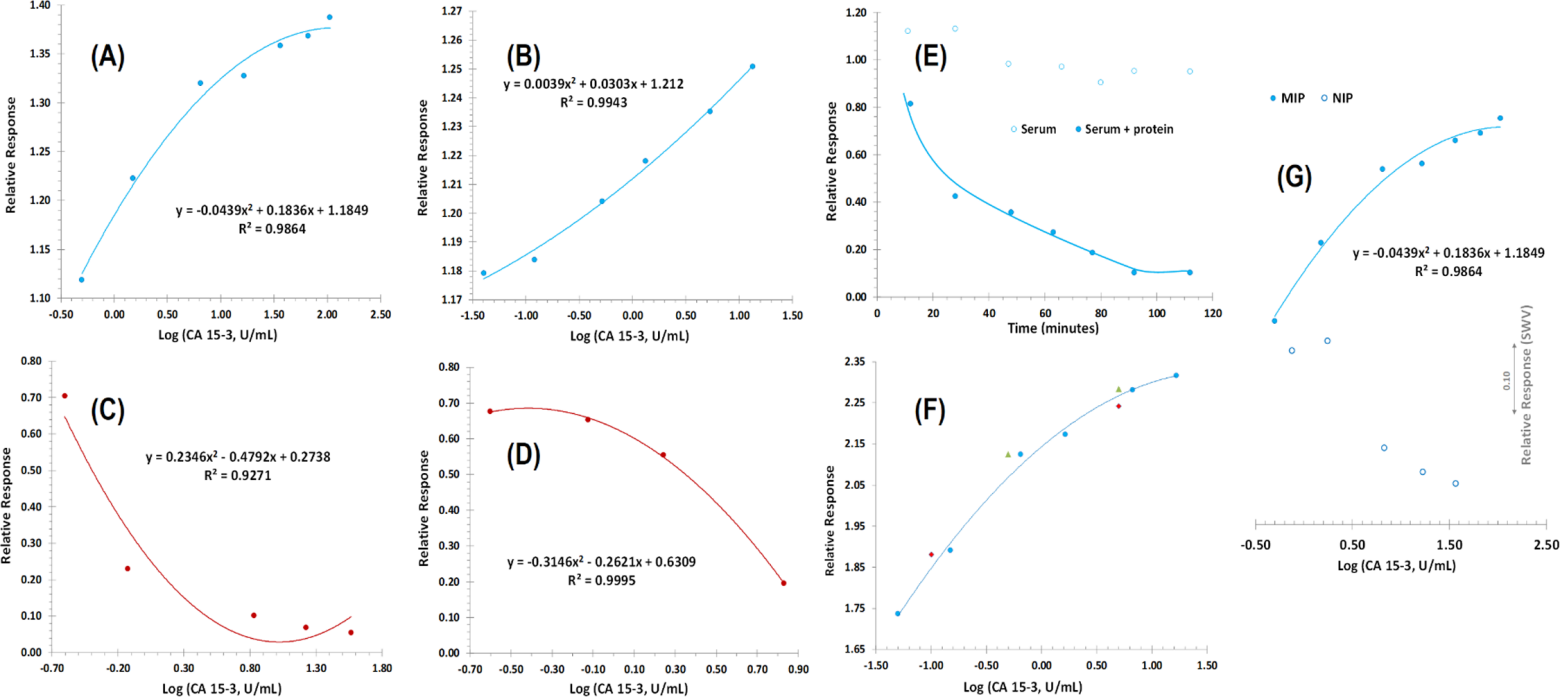
Electrochemical measurements. (A) MIP/–/Au-SPE with charged monomers in PBS buffer; (B) MIP/–/Au-SPE without charged monomers in PBS buffer; (C) NIP/Au-SPE with charged monomers; (D) NIP/Au-SPE without charged monomers in PBS buffer; (E) Comparative temporal response of MIP/Au-SPE with serum and serum+protein incubation; (F) “Blind” comparison MIP/Au-SPE with random protein concentrations; (G) Relative response MIP/–/Au-SPE and NIP/–/Au-SPE in 5.0 mM [Fe(CN)6]^3−^ and 5.0 mM [Fe(CN)6]^4−^, in PBS buffer pH 7.5, with different concentrations of CA15-13.

The effect of the charged monomers was tested by preparing in parallel Au-SPEs with and without charged monomers. The resulting materials were then tested by incubation with CA 15–3 standard solutions prepared in PBS buffer. The obtained calibrations are shown in Fig 8A and 8B, expressed log concentration against the relative values to the signal of the blank. The data were obtained after several incubations in buffer, ensuring a stable electrical reading (typically < 5% variation, in a random behaviour).

In general, the presence of the charged species gave rise to a completely different behaviour of the film in presence of the protein. In SWV data, the current signal decreased by increasing the protein concentration at the surface when the imprinted film had no charged species (Fig 8B). The first incubation in protein dropped the current values significantly, and the last three standards exhibited a little variation of the current, pointing out a tendency for saturation.

When charged species were added into the imprinted polymer, it was curious to observe that the current values increased instead of decreasing (Fig 8A). The profile observed was not a linear trend, as the kinetics seemed to vary along the concentration range tested (0.005 to 40.0 U/mL), even for a log concentration plot, but a mathematical correlation of second degree was established with a good correlation coefficient (~0.99). Further tests were conducted with MIP materials prepared with just one of the charged species to understand this effect (S2 Fig). In general, the less resistive materials were obtained when both charged monomers were present prior to electropolymerization (S2 Fig). The positively charged monomer had a higher resistance than this one and the negatively charged monomer had a much higher resistance than the previous two. Overall, as the incubation of CA 15–3 increases the resistance of the biosensor, the most sensitive system is probably the one starting from a lower charge-transfer resistance value, thereby advising the use of both charged species.

The NIP with charged species had a similar behaviour to the MIP without charges (Fig 8C). This was as expected, because the charged species incubated on the NIP film were covered by the subsequent polymerization of oPDA. Moreover, the trypsin incubation did not change the polymer significantly (no protein was present). Thus, the effect of the interactions of the protein with the external surface of this film should be similar to those of the MIP film prepared without charges.

Moreover, the response to the presence of the protein of the NIP without charges (Fig 8C) was only evidenced for higher concentration levels, compared to the corresponding MIP (Fig 8B). This behaviour confirmed the ability of this MIP to interact with the protein through rebinding sites and that these interactions were dominating the non-specific adsorption within in the lower concentration range of CA 15–3.

Overall, these results pointed-out the relevance of having charged species within the rebinding site and that the MIP alone (with no charges) was able to interact with the protein through the rebinding sites. The difference between the analytical behaviour of the MIP with charges and the MIP without charges (Fig 8G) or even the NIP with charges was highly significant, with the MIP displaying positive slopes after protein incubation and the other biosensors displaying negative slopes under the same condition.

### Ellipsometry studies

The previous material (polymeric oPDA and charged monomers) was further controlled by ellipsometric readings to monitor the layer thickness along the assembly of the biosensor. The obtained data is shown in Table 1. Measurements were made after CA15-3 adsorption on surface, after charged monomers incubated, after electropolymerization and after trypsin removal. An ellipsometric model was created to calculate the response from Fresnel’s equations, which best fitted to the materials thickness and optical constants. Mean Squared Error (MSE) was used as an estimator to quantify the differences between the model and experimental results, with lower MSE values being considered the best ones. As an overall, the data was considered in good fitting to the models employed.

**Table 1:** Thickness of each layer added into the biosensor. The values obtained after each stage of modification of the Au-SPE.

Stage of modification	Thickness (nm)	Mean Squared Error (MSE)
**(1)**, only CA15-3	2.10 ± 0.122	3.247
**(2)**, (1) with Charged Monomers	0.10 ± 0.031	3.487
**(3)**, (2) with oPDA	4.46 ± 8.346	4.665
**(4)**, (3) and Trypsin	0.40 ± 0.048	4.075

The addition of CA 15–3 into the planar gold gave rise to a ~2 nm increase. Since this protein is not a sphere and of huge dimensions, probably it was lying down on the gold support. The subsequent addition of charged monomers yielded a slight increase in the overall thickness; this increase was within the error of the previous layer (CA -15-3), meaning that this value could be of little significance. The addition of the charged monomers (of angstrom size) was most likely not perceptible.

The polymerization of oPDA was the layer imposing a higher variation in the overall thickness. In total, the thickness of the polymer increased by ~4.5 nm, but this value was linked to ~8.3 nm standard deviation. In practice, this layer revealed a roughness of about 16 nm, which is more likely consistent with the size of the natural protein and the cavities left by their exit. It is therefore likely that the protein was just lying down after adsorption (the ~2 nm size on top of the gold) but, when incubated in the oPDA for polymerization, adopted other position and got loose from the underlying surface to be bulked with huge standard deviation is also consistent with the fact that the exit of the protein exposed again the previous gold surface, as confirmed by FTIR and Raman data.

Overall, the ellipsometric studies of the layer thickness changes are more or less consistent with the data obtained by AFM, also supporting previously observed FTIR and Raman analysis.

### Analytical features and application

To be able to have an application of the described biosensor, the MIP biosensor was incubated first in serum and after in increasing concentrations of the target protein prepared in serum. The typical response in EIS is shown in Fig 8E and was plotted against time to represent the consecutive blank incubations. Considering the blank incubations, a slight increase in Rct was observed compared to the initial reading, and then the signal decreased in the same range to reach a random value around 90% of signal corresponding to the initial blank reading. The incubation in serum with a set concentration of CA15-3 had a completely different behaviour. The first time point dropped the Rct levels to about 80% (causing a higher impact than the blank serum) and the values reached a steady signal at about 90 minutes.

The corresponding calibration in SWV is represented in Fig 8G, pointing out a current increase for increasing CA 15–3 concentrations, as reported for calibrations in PBS buffer. The relative change in the current signal was similar to that observed in calibrations with PBS. The NIP response in serum is also shown in Fig 8G, and may be compared in the same graphic with the response of the corresponding MIP, which is also consistent with the calibrations in PBS buffer.

In terms of precision, the standard deviation of consecutive measurements in different calibrations was 5.8% and the repeatability of consecutive readings was less than 5%. Overall, this data confirmed the good precision of this analytical approach proposed herein. Moreover, comparing random concentration values of CA15-3 in MIP/CA15-3/Au-SPE previous calibration curve, a maximum error to 3% was estimated, thereby suggesting the accuracy of this method.

The application feasibility of the biosensor was further tested by preparing spiked serum samples and using a previous calibration curve to extract quantitative data. For this purpose, serum samples were prepared with CA15-3 concentrations (Fig 8F). This analysis was performed in triplicate. Overall, a good agreement was found between added and found amounts of CA15-3 (triangles and diamond points in Fig 8F). The resulting data is shown in Table 2, with recoveries lying within 94 and 97%.

**Table 2:** Comparison between random values of CA15-3 concentration and calibrated values.

Relative Response		C (U/mL)	Relative error (%)
Experimental	Theoretical		
1.89	1.85	0.1	1.85%
2.25	2.27	5.0	1.09%
2.13	2.07	0.5	2.72%
2.28	2.27	5.0	0.73%

Overall, these results seemed promising for direct applications in POC context.

## Conclusions

The effective combination of electrochemistry and molecular imprint technology in an analytical device provided a promising tool for direct electrochemical detection of proteins. This work describes a successful electrosynthesized poly(oPDA) thin film that was molecularly imprinted with CA15-3, and confirms the importance of having charged species with the rebinding position.

In general, the biosensor presented simplicity in designing, short measuring time, high accuracy, ability to detect CA 15–3 within a concentration range of clinical interest, even in diluted serum. The results obtained so far demonstrated that this is a promising method for future practical applications, especially contributing to the screening the breast cancer in POC.

The method here described has the advantage of being of easy manufacture and low cost in relation to conventional methods obtaining results much more quickly and without long waiting times.

## Supporting information

S1 Fig The effect of monomers upon the calibration against CA15-3. SWV measurements of (A) MIP/Au-SPE with phenylenediamine as a monomer; (B) MIP/Au-SPE with aniline and corresponding calibration curve. 5.0 mM [Fe(CN)6]^3−^ and 5.0 mM [Fe(CN)6]^4−^, in PBS buffer pH 7.5, with different concentrations of CA15-3.
(TIF)

S2 Fig Comparison between MIP with both charged species. SA (-) and DPM (+) and MIP with just one of the species present. (A) Electropolymerization (B) FRA (C) CV, FRA and CV assessed in 5.0 mM [Fe(CN)_6_]^3−^ and 5.0 mM [Fe(CN)_6_]^4−^, in PBS buffer, pH 7.
(TIF)

## References

[1] Duffy MJ, Evoy D, McDermott EW. CA 15–3: uses and limitation as a biomarker for breast cancer. Clinica chimica acta; international journal of clinical chemistry. 2010;411(23–24):1869–74. doi: 10.1016/j.cca.2010.08.039 . 20816948 10.1016/j.cca.2010.08.039

[2] Wang J. Electrochemical biosensors: towards point-of-care cancer diagnostics. Biosensors & bioelectronics. 2006;21(10):1887–92. doi: 10.1016/j.bios.2005.10.027 . 16330202 10.1016/j.bios.2005.10.027

[3] Wu J, Zhang Z, Fu Z, Ju H. A disposable two-throughput electrochemical immunosensor chip for simultaneous multianalyte determination of tumor markers. Biosensors & bioelectronics. 2007;23(1):114–20. doi: 10.1016/j.bios.2007.03.023 . 17475473 10.1016/j.bios.2007.03.023

[4] Rasooly A, Jacobson J. Development of biosensors for cancer clinical testing. Biosensors & bioelectronics. 2006;21(10):1851–8. doi: 10.1016/j.bios.2006.01.003 . 16458498 10.1016/j.bios.2006.01.003

[5] Wu J, Fu Z, Yan F, Ju H. Biomedical and clinical applications of immunoassays and immunosensors for tumor markers. TrAC Trends in Analytical Chemistry. 2007;26(7):679–88. doi: 10.1016/j.trac.2007.05.007

[6] Tothill IE. Biosensors for cancer markers diagnosis. Seminars in cell & developmental biology. 2009;20(1):55–62. doi: 10.1016/j.semcdb.2009.01.015 . 19429492 10.1016/j.semcdb.2009.01.015

[7] Colomer R, Ruibal A, Genolla J, Salvador L. Circulating CA 15–3 antigen levels in non-mammary malignancies. British journal of cancer. 1989;59(2):283–6. 2930693 10.1038/bjc.1989.58 PMC2246988

[8] Soletormos G, Nielsen D, Schioler V, Mouridsen H, Dombernowsky P. Monitoring different stages of breast cancer using tumour markers CA 15–3, CEA and TPA. European journal of cancer. 2004;40(4):481–6. doi: 10.1016/j.ejca.2003.10.015 . 14962712 10.1016/j.ejca.2003.10.015

[9] Mohammadnejad J, Rasaee MJ, Saqhafi B, Rajabibazl M, Rahbarizadeh F, Omidfar K, et al A new competitive enzyme linked immunosorbent assay (MRP83-CA15-3) for MUC1 measurement in breast cancer. Journal of Immunoassay & Immunochemistry. 2006;27(2):139–49. doi: 10.1080/15321810600573077 PubMed PMID: WOS:000237300100003. 16711252 10.1080/15321810600573077

[10] Abe Y, Odagiri E, Jibiki K, Demura R, Demura H. A study of the clinical usefulness of the CA15-3 radioimmunoassay kit. Gan no rinsho Japan journal of cancer clinics. 1988;34(12):1672–6. PubMed PMID: MEDLINE:3193612. 3193612

[11] Shi M, Zhao S, Huang Y, Liu Y-M, Ye F. Microchip fluorescence-enhanced immunoaasay for simultaneous quantification of multiple tumor markers. Journal of Chromatography B-Analytical Technologies in the Biomedical and Life Sciences. 2011;879(26):2840–4. doi: 10.1016/j.jchromb.2011.08.013 PubMed PMID: WOS:000295297800023. 21873123 10.1016/j.jchromb.2011.08.013

[12] Liu Y-M, Zheng Y-L, Cao J-T, Chen Y-H, Li F-R. Sensitive detection of tumor marker CA15-3 in human serum by capillary electrophoretic immunoassay with chemiluminescence detection. Journal of Separation Science. 2008;31(6–7):1151–5. doi: 10.1002/jssc.200700590 PubMed PMID: WOS:000255613800032. 18335473 10.1002/jssc.200700590

[13] Grzywa R, Lupicka-Slowik A, Walczak M, Idzi M, Bobrek K, Boivin S, et al Highly sensitive detection of cancer antigen 15–3 using novel avian IgY antibodies. Altex. 2014;31(1):43–52. doi: 10.14573/altex.1309181 . 24270753 10.14573/altex.1309181

[14] He ZH, Jin WR. Electrochemical enzyme immunoassay of tumor marker CA15-3 with capillary electrophoresis. Chinese Chemical Letters. 2002;13(11):1090–2. PubMed PMID: WOS:000179252700020.

[15] Zhang XL, Peng XW, Jin WR. Scanning electrochemical microscopy with enzyme immunoassay of the cancer-related antigen CA15-3. Analytica Chimica Acta. 2006;558(1–2):110–4. doi: 10.1016/j.aca.2005.11.032 PubMed PMID: WOS:000235116600018.

[16] Wang G, Qing Y, Shan J, Jin F, Yuan R, Wang D. Cation-exchange antibody labeling for simultaneous electrochemical detection of tumor markers CA15-3 and CA19-9. Microchimica Acta. 2013;180(7–8):651–7. doi: 10.1007/s00604-013-0973-z PubMed PMID: WOS:000319071300016.

[17] Lin J, Ju H. Electrochemical and chemiluminescent immunosensors for tumor markers. Biosensors & bioelectronics. 2005;20(8):1461–70. doi: 10.1016/j.bios.2004.05.008 . 15626599 10.1016/j.bios.2004.05.008

[18] Sharma PS, Pietrzyk-Le A, D'Souza F, Kutner W. Electrochemically synthesized polymers in molecular imprinting for chemical sensing. Analytical and Bioanalytical Chemistry. 2012;402(10):3177–204. doi: 10.1007/s00216-011-5696-6 PubMed PMID: WOS:000301542200013. 22302165 10.1007/s00216-011-5696-6 PMC3303047

[19] Chen L, Xu S, Li J. Recent advances in molecular imprinting technology: current status, challenges and highlighted applications. Chemical Society Reviews. 2011;40(5): 2922–2942. doi: 10.1039/c0cs00084a PubMed PMID: WOS:000289630700046. 21359355 10.1039/c0cs00084a

[20] Turner NW, Jeans CW, Brain KR, Allender CJ, Hlady V, Britt DW. From 3D to 2D: A review of the molecular imprinting of proteins. Biotechnology Progress. 2006;22(6):1474–89. doi: 10.1021/bp060122g PubMed PMID: WOS:000242427200002. 17137293 10.1021/bp060122g PMC2666979

[21] Frasco MF, Truta LA, Sales MG, Moreira FT. Imprinting Technology in Electrochemical Biomimetic Sensors. Sensors (Basel). 2017;17(3). doi: 10.3390/s17030523 ; PubMed Central PMCID: PMC5375809. 28272314 10.3390/s17030523 PMC5375809

[22] Piletsky SA, Piletska EV, Chen BN, Karim K, Weston D, Barrett G, et al Chemical grafting of molecularly imprinted homopolymers to the surface of microplates. Application of artificial adrenergic receptor in enzyme-linked assay for beta-agonists determination. Analytical Chemistry. 2000;72(18):4381–5. doi: 10.1021/ac0002184 PubMed PMID: WOS:000089308300017. 11008773 10.1021/ac0002184

[23] Titirici MM, Sellergren B. Thin molecularly imprinted polymer films via reversible addition-fragmentation chain transfer polymerization. Chemistry of Materials. 2006;18(7). doi: 10.1021/cm052153x PubMed PMID: WOS:000236499300009.

[24] Henry OYF, Piletsky SA, Cullen DC. Fabrication of molecularly imprinted polymer microarray on a chip by mid-infrared laser pulse initiated polymerisation. Biosensors & Bioelectronics. 2008;23(12). doi: 10.1016/j.bios.2008.02.010 PubMed PMID: WOS:000256736300002. 18378439 10.1016/j.bios.2008.02.010

[25] Katz A, Davis ME. Molecular imprinting of bulk, microporous silica. Nature. 2000;403(6767):286–9. PubMed PMID: WOS:000084899700045. doi: 10.1038/35002032 10659842 10.1038/35002032

[26] Reddy SM, Sette G, Phan Q. Electrochemical probing of selective haemoglobin binding in hydrogel-based molecularly imprinted polymers. Electrochimica Acta. 2011;56(25):9203–8. doi: 10.1016/j.electacta.2011.07.132 PubMed PMID: WOS:000295997000028.

[27] Rimmer S. Synthesis of molecular imprinted polymer networks. Chromatographia. 1998;47(7–8):470–4. PubMed PMID: WOS:000073458400020.

[28] Moreira FTC, Dutra RAF, Noronha JPC, Sales MGF. Myoglobin-biomimetic electroactive materials made by surface molecular imprinting on silica beads and their use as ionophores in polymeric membranes for potentiometric transduction. Biosensors & Bioelectronics. 2011;26(12):4760–6. doi: 10.1016/j.bios.2011.05.045 PubMed PMID: WOS:000293932300019. 21683568 10.1016/j.bios.2011.05.045

[29] Moreira FTC, Dutra RAF, Noronha JPC, Cunha AL, Sales MGF. Artificial antibodies for troponin T by its imprinting on the surface of multiwalled carbon nanotubes: Its use as sensory surfaces. Biosensors & Bioelectronics. 2011;28(1):243–50. doi: 10.1016/j.bios.2011.07.026 PubMed PMID: WOS:000295661700037. 21816602 10.1016/j.bios.2011.07.026

[30] Zdyrko B, Hoy O, Luzinov I. Toward protein imprinting with polymer brushes. Biointerphases. 2009;4(2). doi: 10.1116/1.3101907 PubMed PMID: WOS:000266976300003. 20408713 10.1116/1.3101907

[31] Zayats M, Kanwar M, Ostermeier M, Searson PC. Molecular Imprinting of Maltose Binding Protein: Tuning Protein Recognition at the Molecular Level. Macromolecules. 2011;44(10):3966–72. doi: 10.1021/ma200355j PubMed PMID: WOS:000290511500036.

[32] Cabral-Miranda G, Gidlund M, Sales MGF. Backside-surface imprinting as a new strategy to generate specific plastic antibody materials. Journal of Materials Chemistry B. 2014;2(20):3087 doi: 10.1039/c3tb21740j 10.1039/c3tb21740j 32261684

[33] Li J, Zhao J, Wei X. A sensitive and selective sensor for dopamine determination based on a molecularly imprinted electropolymer of o-aminophenol. Sensors and Actuators B-Chemical. 2009;140(2):663–9. doi: 10.1016/j.snb.2009.04.067 PubMed PMID: WOS:000268215200048.

[34] Choong CL, Bendall JS, Milne WI. Carbon nanotube array: A new MIP platform. Biosensors & Bioelectronics. 2009;25(3):652–6. doi: 10.1016/j.bios.2008.11.025 PubMed PMID: ISI:000271931900020. 19162461 10.1016/j.bios.2008.11.025

[35] Apodaca DC, Pernites RB, Ponnapati RR, Del Mundo FR, Advincula RC. Electropolymerized Molecularly Imprinted Polymer Films of a Bis-Terthiophene Dendron: Folic Acid Quartz Crystal Microbalance Sensing. Acs Applied Materials & Interfaces. 2011;3(2):191–203. doi: 10.1021/am100805y PubMed PMID: WOS:000287639400016. 21080660 10.1021/am100805y

[36] Wu S, Tan W, Xu H. Protein molecularly imprinted polyacrylamide membrane: for hemoglobin sensing. Analyst. 2010;135(10):2523–7. doi: 10.1039/c0an00191k PubMed PMID: WOS:000282003800005. 20625585 10.1039/c0an00191k

[37] Stejskal J. Polymers of phenylenediamines. Progress in Polymer Science. 2015;41:1–31. doi: 10.1016/j.progpolymsci.2014.10.007

[38] Sayyah SM, El-Rabiey MM, Abd El-Rehim SS, Azooz RE. Electropolymerization kinetics of o-aminophenol and characterization of the obtained polymer films. Journal of Applied Polymer Science. 2006;99(6):3093–109. doi: 10.1002/app.22915 PubMed PMID: WOS:000235343500030.

[39] Tucceri R. A Review About the Charge Conduction Process at Poly(o-aminophenol)Film Electrodes. The Open Physical Chemistry Journal; 2010; 62–77.

[40] Moreira FTC, Dutra RAF, Noronha JPC, Fernandes JCS, Sales MGF. Novel biosensing device for point-of-care applications with plastic antibodies grown on Au-screen printed electrodes. Sensors and Actuators B: Chemical. 2013;182:733–40. doi: 10.1016/j.snb.2013.03.099

[41] Daniels JS, Pourmand N. Label-free impedance biosensors: Opportunities and challenges. Electroanalysis. 2007;19(12):1239–57. doi: 10.1002/elan.200603855 PubMed PMID: WOS:000247489500001. 18176631 10.1002/elan.200603855 PMC2174792

[42] Suni II. Impedance methods for electrochemical sensors using nanomaterials. Trac-Trends in Analytical Chemistry. 2008;27(7):604–11. doi: 10.1016/j.trac.2008.03.012 PubMed PMID: WOS:000258747400016.

[43] Xiao-Zi Yuan CS, Haijiang Wang, Jiujun Zhang. Electrochemical Impedance Spectroscopy in PEM Fuel Cells Fundamentals and Applications.: Springer-Verlag London; 2010 420 p.

[44] Levin O, Kondratieva V, Malev V. Charge transfer processes at poly-o-phenylenediamine and poly-o-aminophenol films. Electrochimica Acta. 2005;50(7–8):1573–85. doi: 10.1016/j.electacta.2004.10.028 PubMed PMID: WOS:000226969600017.

[45] Malitesta C, Losito I, Zambonin PG. Molecularly imprinted electrosynthesized polymers: New materials for biomimetic sensors. Analytical Chemistry. 1999;71(7):1366–70. doi: 10.1021/ac980674g PubMed PMID: WOS:000079593900031. 21662960 10.1021/ac980674g

[46] Boyer MI, Quillard S, Louarn G, Froyer G, Lefrant S. Vibrational study of the FeCl3-doped dimer of polyaniline; A good model compound of emeraldine salt. Journal of Physical Chemistry B. 2000;104(38):8952–61. doi: 10.1021/jp000946v PubMed PMID: WOS:000089561400008.

[47] Trchova M, Moravkova Z, Blaha M, Stejskal J. Raman spectroscopy of polyaniline and oligoaniline thin films. Electrochimica Acta. 2014;122:28–38. doi: 10.1016/j.electacta.2013.10.133 PubMed PMID: WOS:000334007900006.

[48] do Nascimento GM, Temperini MLA. Studies on the resonance Raman spectra of polyaniline obtained with near-IR excitation. Journal of Raman Spectroscopy. 2008;39(7):772–8. doi: 10.1002/jrs.1841 PubMed PMID: WOS:000258076200002.

[49] Ciri-Marjanovic G, Trchova M, Stejskal J. The chemical oxidative polymerization of aniline in water: Raman spectroscopy. Journal of Raman Spectroscopy. 2008;39(10):1375–87. doi: 10.1002/jrs.2007 PubMed PMID: WOS:000260355900010.

