# Supplementary figures and images for: Sensing CA 15-3 in point-of-care by electropolymerizing O-phenylenediamine (oPDA) on Au-screen printed electrodes
Source: PLoS One. 2018 May 1;13(5):e0196656. doi: 10.1371/journal.pone.0196656 (PMC5929556; doi:10.1371/journal.pone.0196656)

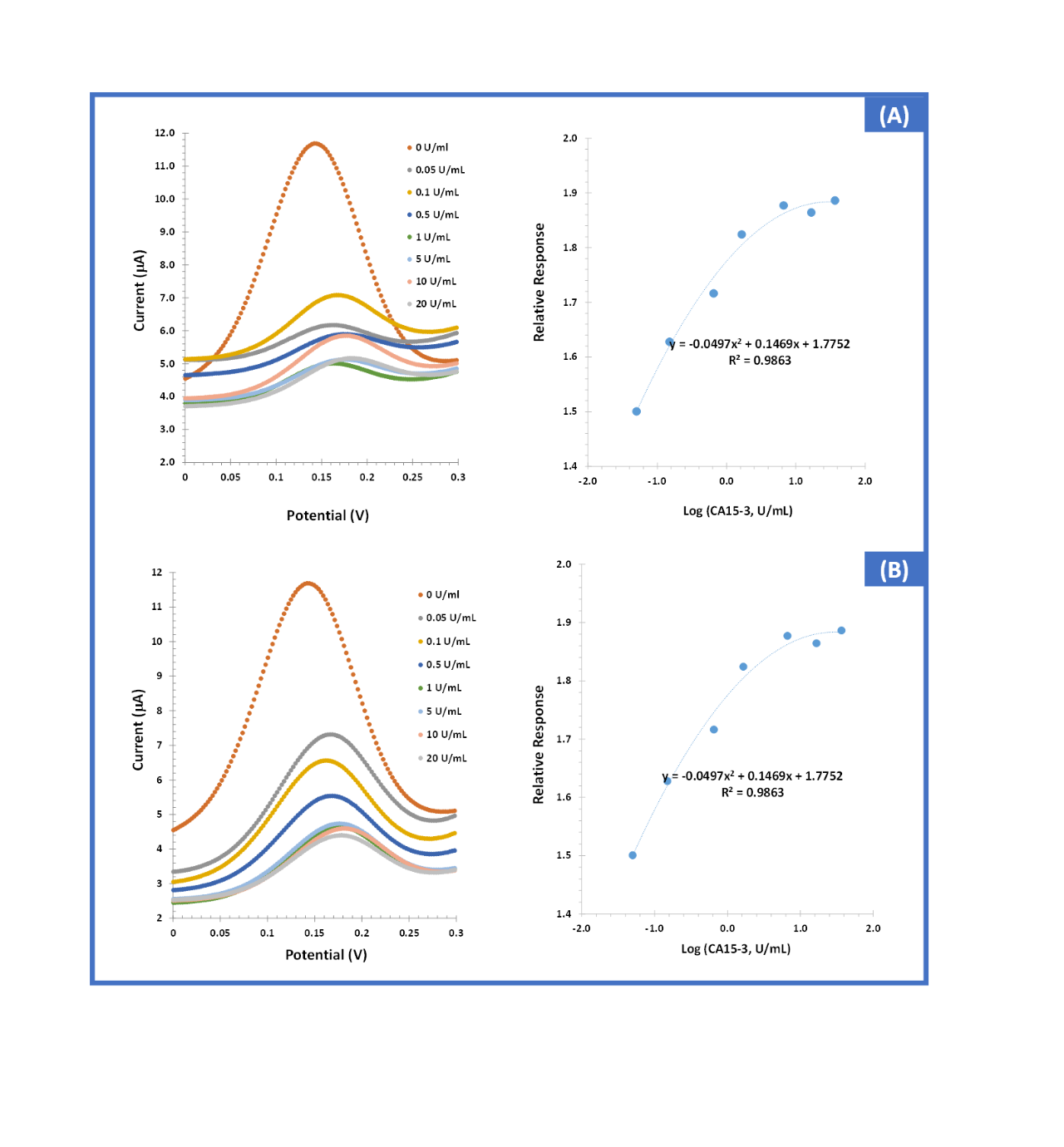

Supplement: S1 Fig — SWV measurements of (A) MIP/Au-SPE with phenylenediamine as a monomer; (B) MIP/Au-SPE with aniline and corresponding calibration curve. 5.0 mM [Fe(CN)6]3− and 5.0 mM [Fe(CN)6]4−, in PBS buffer pH 7.5, with different concentrations of CA15-3. (TIF) [file pone.0196656.s001.tif]

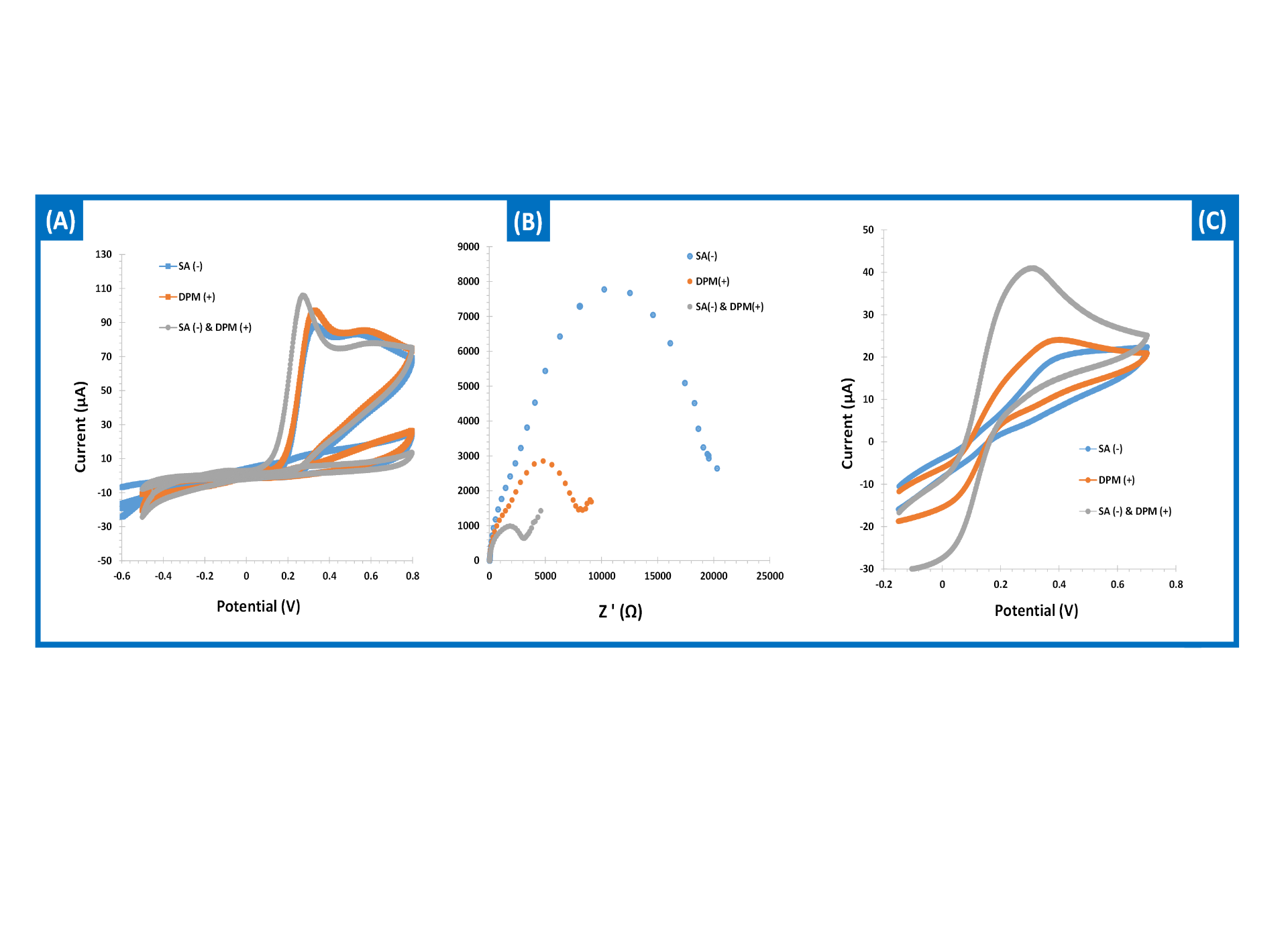

Supplement: S2 Fig — SA (-) and DPM (+) and MIP with just one of the species present. (A) Electropolymerization (B) FRA (C) CV, FRA and CV assessed in 5.0 mM [Fe(CN)6]3− and 5.0 mM [Fe(CN)6]4−, in PBS buffer, pH 7. (TIF) [file pone.0196656.s002.tif]
